# Mapping Wide Row Crops with Video Sequences Acquired from a Tractor Moving at Treatment Speed

**DOI:** 10.3390/s110707095

**Published:** 2011-07-11

**Authors:** Nadir Sainz-Costa, Angela Ribeiro, Xavier P. Burgos-Artizzu, María Guijarro, Gonzalo Pajares

**Affiliations:** 1 Centre for Automation and Robotics, CSIC-UPM, Arganda del Rey, 28500 Madrid, Spain; E-Mail: nadir.sainz@car.upm-csic.es; 2 Computation and Neural Systems, 136-93, California Institute of Technology, 1200 East California Boulevard, Pasadena, CA 91125, USA; E-Mail: xpburgos@gmail.com; 3 Department of Software Engineering and Artificial Intelligence, Faculty of Computer Science, Complutense University, 28040 Madrid, Spain; E-Mails: mguijarro@fdi.ucm.es (M.G.); pajares@fdi.ucm.es (G.P.)

**Keywords:** crop mapping, video stabilization, inverse perspective mapping, video processing, precision agriculture

## Abstract

This paper presents a mapping method for wide row crop fields. The resulting map shows the crop rows and weeds present in the inter-row spacing. Because field videos are acquired with a camera mounted on top of an agricultural vehicle, a method for image sequence stabilization was needed and consequently designed and developed. The proposed stabilization method uses the centers of some crop rows in the image sequence as features to be tracked, which compensates for the lateral movement (sway) of the camera and leaves the pitch unchanged. A region of interest is selected using the tracked features, and an inverse perspective technique transforms the selected region into a bird’s-eye view that is centered on the image and that enables map generation. The algorithm developed has been tested on several video sequences of different fields recorded at different times and under different lighting conditions, with good initial results. Indeed, lateral displacements of up to 66% of the inter-row spacing were suppressed through the stabilization process, and crop rows in the resulting maps appear straight.

## 1. Introduction

Precision Agriculture aims to optimize field management and increase agricultural efficiency and sustainability; that is, to reduce the operating costs and ecological footprint traditionally associated with agriculture by matching resource application and agronomic practices with soil and crop requirements.

For instance, most herbicides are usually applied uniformly in fields, but strong evidence suggests that weeds occur in patches rather than in homogenous distributions within crop fields. Marshall *et al.* investigated the presence of three different species of grass in arable fields and showed that between 24% and 80% of the sample area was free of grass weeds [1]. According to [2], an average of 30% of the sample area for 12 fields (seven maize and five soybean fields) was free of broadleaf weeds, and 70% was free of grass weeds in the inter-row spacing where no herbicide was previously applied. In these situations, accurate maps showing both weed location and density could have (and indeed have had) numerous uses including monitoring the effectiveness of past or current weed management strategies, understanding weed population dynamics and verifying model predictions. In particular, they can be the data source for sprayers, which can determine their location using a GPS receiver and apply treatments where data recommends it. These spatial information systems have the potential to allow farmers to fine-tune the locations and rates of herbicide application, thereby achieving sustainability and reducing treatment costs [3]. In [3,4] the authors report that by using site-specific weed control, reductions of between 42% (soybean and maize) and 84% (maize) in the amount of applied herbicide could be achieved, depending on the patchiness and weed pressure in the sample fields. These herbicide savings translated into an average of 33 €/ha per year that would be available to apply to the additional costs for sampling, data processing and precision spraying [5].

Two main approaches to the data-collection step exist: sampling from the air and from ground level. Aerial imagery and satellite data lack the necessary spatial resolution, and their acquisition depends heavily on weather conditions (e.g., lack of clouds and fog). In the mid-late’90 these aerial methods fell in disuse due to the appearance of more advanced computers that permitted direct photo analysis, though nowadays are experiencing a resurgence due to the use of hyper and multispectral cameras, that facilitate and potentiate the reckoning of each species [6]. Still, these methods continue to show clear disadvantages like their high economic costs and low resolution due to the height from which images is taken, causing each pixel to represent more than a square meter of area.

At the ground level, data collection can be accomplished by sampling on foot or with mobile platforms. Sampling on foot is a highly time-consuming task and requires a high number of skilled workers to cover the large treatment areas and even doing so, only discrete data are obtained (using sampling grids) [7]. Colliver *et al.* calculated the time needed to manually map the presence of wild oats in a field as 3.75 h/ha [8]. Depending on the size of the sampling grid, the required time can vary between 4.36 h/ha for a 20 m × 20 m grid [9] and 2.5 h/ha using a 36 m × 50 m grid [10]. Thus, the cost of manually mapping the weeds in a field would exceed the savings gained from the reduced herbicide use.

On the other hand, data gathering using a tractor or vehicle as a mobile platform requires only one operator and enables continuous sampling. In continuous sampling, data are collected over the entire sample area, whereas with discrete sampling, data are collected only from pre-defined points throughout an area. Interpolation methods are then used to estimate the densities in the intervening areas. Continuous data can provide a qualitative description of abundance (*i.e*., presence or absence, or zero, low, medium, or high) rather than the quantitative plant counts usually generated from discrete sampling [1]. Moreover, acquiring video from a mobile platform may become a good opportunity to obtain accurate weed and crop maps, which is our objective in this paper, and also crop row location in real time has often been an important goal in the autonomous guidance of agricultural vehicles [11], which increments the advantages of the ground level approach.

Mounting cameras on top of tractors or mobile platforms presents problems because the roughness of the terrain transfers to the camera mounting system and causes it to acquire images that are difficult to process (even to the human eye). Image sequence stabilization is the process of removing (totally or partially) the effects of this unwanted motion from an input video sequence. It is a key pre-processing step in any serious application of computer vision, especially when images are acquired from a mobile platform. Based on the particular roughness of the terrain, motion in Precision Agriculture video sequences can include vibration, sway, roll and pitch.

**Figure 1.**
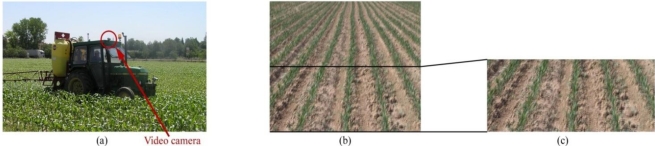
**(a)** The emplacement of the video camera onboard the tractor; **(b)** The original crop field image. Closer to the corners, crop rows become difficult to distinguish from one another; **(c)** Half of the original image, where crop rows are clearly identifiable.

The problem of image stabilization has been assessed by a number of researchers [12–18]. Different techniques are used in the literature and are primarily based on sparse feature tracking; some of them use Kalman filters to predict the motion of features from frame to frame [12]. The authors of [13] use Kalman filters to estimate the camera velocity and acceleration vectors for each frame using linear camera motion models. Both [14] and [15] estimate the optical flow field and compute the required affine transformation using Laplacian pyramid images. In [16], the 3D motion of the camera is computed by first calculating the image flow of a 2D planar region and then subtracting it from the whole image flow, resulting in a rotationless optical flow. This rotationless image sequence is considered to be stabilized for relevant purposes. In [17], almost vertical segments in the frames are used to compute the transformation needed to make them *real* vertical lines and thus correct the camera rotation. In [18] block motion vectors are used to estimate the motion between consecutive frames. These methods attempt to compensate for all motion and are rather complex and computationally demanding. Most of them use discrete features (*i.e*., corners or segments) and attempt to keep these features fixed in relation to a reference frame. These solutions are not possible in our case, where there are no permanent features or even a constant reference frame, because the portion of the field recorded by the camera is constantly changing as the vehicle travels through it. In the context of wide row crops (see Figure 1(b)), we can exploit some characteristics of the images, particularly the fact that crop fields present an approximately constant pattern of evenly spaced parallel rows. In this paper, we present a method to stabilize the sway and roll motion in crop field video sequences using the crop rows as features and inverse perspective mapping to focus on a region of interest. This method is implemented as a first step in the mapping of crop fields using OpenCV [19].

After stabilization, a map containing all vegetation cover (crop rows and weeds) was built. In this map, crop rows are quite straight regardless of the camera movements that, without stabilization, would make them appear as S-shaped lines. Generation of weed maps has been reported in the literature. In [20], weeds are mapped automatically using three bi-spectral cameras mounted in front of a prototype carrier vehicle. This is a rather expensive system due to the specialized cameras and the dedicated mobile platform. The idea behind our proposal is to use a good quality domestic camera mounted on top of an agricultural vehicle that is likely dedicated to some other field task and therefore presents a more cost effective solution. Tian *et al.* developed a real-time precision spraying system that releases herbicide only over weed patches based on the information gathered by two to four cameras mounted in front of the sprayer. Instead of building a weed map, the system acts in real-time [3]. Although this presents certain advantages, we argue that the information contained in a map is a very powerful tool because it can be used to measure the effectiveness of the treatments from season to season, to extract global information about weed coverage in the field or to distribute tasks among a robot fleet.

Unfortunately, none of these studies thus far have resulted in the commercialization of the technologies developed. The major obstacles to commercialization concern the high computing and economic costs involved, as well as the difficulties of correctly representing all of the possible situations present in real and outdoor conditions [21].

## 2. Materials and Methods

### 2.1. Frame Segmentation Process

All frames used for presenting and testing our proposal have a 720 × 576 pixel resolution and were taken with a commercial video camera (Sony DCR PC110E) that was placed directly on the roof of the tractor, at a height of 2.15 m from the ground, with a 10° pitch angle [Figure 1(a)]. The images were acquired during a treatment operation at an approximate speed of 6 km/h. In the crop images, our interest focused on the central three rows because they are present in every frame (even when the camera sways laterally) and they can be seen with moderate resolution. Closer to the upper corners of the frame, the crop rows become difficult to distinguish from one another due to the perspective in the image and the camera optics [as seen in Figure 1(b)]. To avoid these effects, the image was divided in half, and the upper half was discarded [Figure 1(c)].

**Figure 2.**
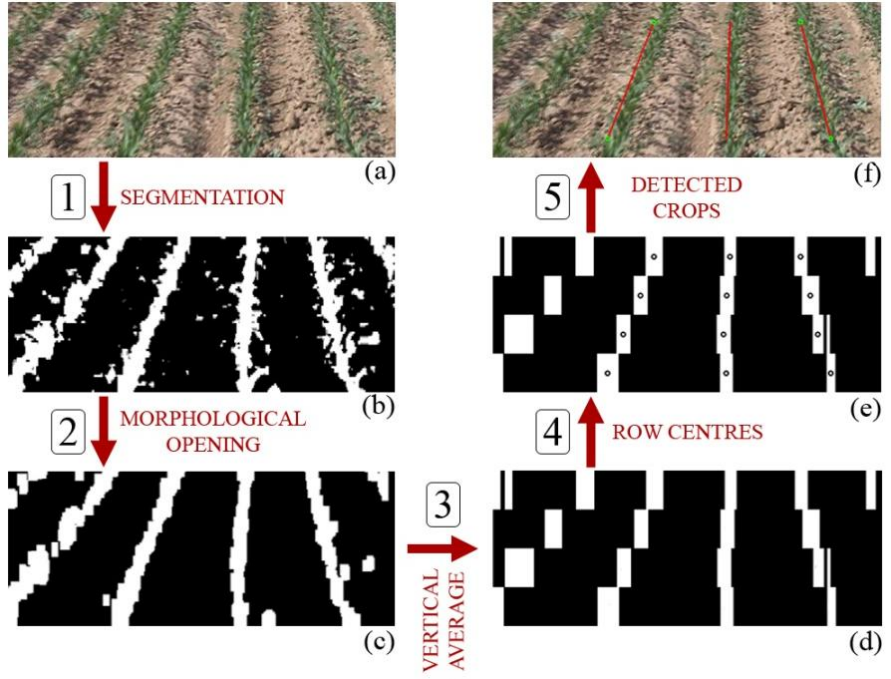
**(a)** Original RGB image of a wide row-crop field before any processing. Crop rows are clearly identifiable; **(b)** The same frame after the segmentation process is applied to the RGB image. Crops rows and weeds are present (white pixels); **(c)** Sample image after morphological operations are applied. Crop rows are denser, and the weed presence has been reduced and disconnected from the crop row; **(d)** Image showing the vertical average of the pixel values for each strip in a given frame of the sequence, once a threshold is applied to eliminate darker grey tones; **(e)** Calculated average centers for the same selected frame of the video sequence; **(f)** Original RGB frame with the average centers and calculated lines.

The first step in the proposed process segments vegetation cover against the background. Therefore it converts the input RGB image into a binary (black and white) image and showing vegetation (crop rows and weeds) in white and the rest as black pixels (Step 1 in Figure 2). Procedures for the segmentation of vegetation pixels usually make use of the fact that pixels belonging to vegetation have stronger green components than any other color. This feature can be used to create a color index that represents how green a certain pixel is [22,23]. The color frame can be transformed into a grayscale (monochrome) image by means of a linear combination of the red, green and blue planes as described in Equation (1):

(1) $$\begin{array}{ll} {\mathrm{image}}_{\mathrm{gray}}(i,j)= & r*{\mathrm{image}}_{\mathrm{red}}(i,j)+g*{\mathrm{image}}_{\mathrm{green}}(i,j)+b*{\mathrm{image}}_{\mathrm{blue}}(i,j)\\ & \forall i\in \mathrm{rows}\_\mathrm{image}\wedge \forall j\in \mathrm{columns}\_\mathrm{image} \end{array}$$

In Equation (1), *image_red_* (*i*, *j*), *image_green_* (*i*, *j*), and *image_blue_* (*i*, *j*) are the red, green and blue 0–255 intensities at pixel (*i*,*j*) respectively, and *r*, *g* and *b* are real coefficients that determine the construction of the monochrome image. These values are crucial in the segmentation of vegetation against the background, and their selection is discussed in the literature [22–25]. Here, they were set using the coefficients proposed in [25], *r =* −0.884, *g =* 1.262, and *b =* −0.311. These coefficients were determined using a genetic algorithm optimization [24] and were proved to perform better than the Excess Green coefficients (*r* = −1, *g* = 2, *b* = −1) given in [22].

A threshold function can convert a grayscale image into a binary image in which white pixels correspond to vegetation and black pixels to the rest. A sample frame of the result of this process is shown in Figure 2(b). Because our only goal at this stage is to track the crop rows, and due to the weeds contained in the binary images, the images need further processing to remove the weeds and to disconnect them from the crop rows. This can be accomplished by applying two morphological operations, namely erosion and dilation. The former eliminates isolated white pixels, and the latter expands those areas where white pixels are dense (Step 2 in Figure 2). Erosion and dilation use different structuring elements in this case: erosion intends to eliminate small weed areas and thus uses a slightly vertical structuring element (10 × 15 pixel rectangular shape), and dilation makes white crop rows denser and eliminates breaks using a vertical 10 × 20 pixel rectangular shape. This morphological opening transforms the binary images containing crop rows and weeds into images where the crop rows stand out [as shown in Figure 2(c)].

### 2.2. Crop Row Tracking Algorithm

The segmentation process results in a binary image containing only a certain number of white crop rows against a black background and some residual white areas due to weeds. To stabilize the lateral sway of the camera, selecting and then tracking some features in every frame is necessary to calculate the compensation needed to make the image sequence steady.

Due to the height and angle of the camera, the frames in the recorded video sequences have no horizon line; therefore, that feature cannot be used to stabilize the video sequences. However, the crop rows are present in every frame of the video sequence and are ideal tracking candidate elements. Consequently, a certain number of crop-row centers at fixed y-coordinates of the image are chosen. The lower half of the frame is divided into four strips of equal height. Then, the vertical centers of those strips are selected as the y-coordinates of the points to be tracked. The x-coordinates of these points are calculated as the average horizontal centers of the crop rows in those strips.

We added all pixel intensity values (0 or 255) for every column in every frame strip and divided that total column value by the strip height. This yielded an average intensity for every column of the strip that corresponds to a certain gray level. The darkness of this gray level indicates the vegetation content of that column: darker columns indicate lower vegetation content, and lighter ones indicate higher vegetation content. Because we need to separate the crop rows (highest vegetation content, close to 100 %) from the rest (weeds and soil with little or no vegetation presence), it seems adequate to apply a threshold on the resulting image (Step 3 in Figure 2). This generates a binary image in which the widest white blocks correspond to the crop rows and the narrower ones (if any) correspond to weed patches that seldom extend over any appreciable vertical distance [Figure 2(d)].

The algorithm uses these wide or narrow characteristics of the white blocks to classify them as crop rows or weeds and then extracts the x-coordinates from the centers of the three central wide blocks, which correspond to crop rows (Step 4 in Figure 2). In the first frame, the algorithm searches for these centers in a window around some known positions (the horizontal center of the image ± the approximated row distance in the image of 140 pixels) and stores them in an array. These centers are distributed over the three central rows of crops [as shown in Figure 2(e)]. Line equations (slope and intercept) are calculated for these three rows (Step 5 in Figure 2) and can be seen in Figure 2(f).

The straightforward feature tracking mechanism stores the crop-row centers in an array after the first frame calculations. A new frame is then processed similarly but using the stored centers from the previous frame as the origins around which the system searches for new crop-row centers. This enables the algorithm to search for a given center only in a window of a certain width around the last known position of that same center. Furthermore the system can find the centers of the same crop rows in every frame even if they move laterally from frame to frame. To a certain extent, abrupt feature displacements from one frame to the next may disable the algorithm from finding the same feature in subsequent frames. However, frame to frame displacements are generally small, given that video sequences recording at 25 fps (or 40 ms per frame) generate them.

### 2.3. Inverse Perspective Mapping

**Figure 3.**
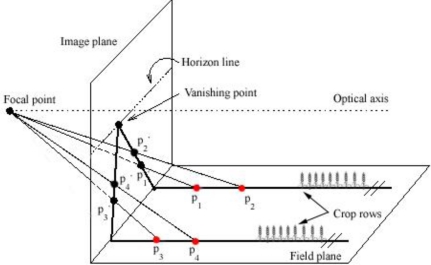
Geometry of the central projective model. Parallel crop rows in the field plane are not parallel and intersect at the vanishing point in the image plane.

The images taken with cameras are 2D projections of the 3D world, and the recovery of 3D information such as depth, length or area requires a model of the projection transformation. The correct model for human vision and cameras is the central projective model (or perspective). Images formed under this model disable the calculation of distance measurements because perspective is a non-linear transformation. Light rays passing through one unique point (the focal point) form the projected image [26]. Figure 3 shows the geometry of this perspective projection. A point (**p1**) belonging to a crop row in the horizontal plane was projected onto the image plane following a line through the focal point. Other points (**p2**, **p3** and **p4**) in the same row, as well as in a parallel crop row, were projected in the same way. All these points belong to parallel crop rows in the field plane, but these rows are not parallel in the image plane due to the non-linear aspects of perspective. Removing these perspective effects and recover parallel lines requires the application of inverse perspective mapping.

Inverse perspective mapping is widely discussed in the literature [26–29]. By using homogeneous coordinates, the non-linear perspective mapping can be expressed as a linear transformation between two planes (*planar homography*), namely the field (horizontal) plane and the camera (image) plane. According to [27], a point (**P**) with homogeneous coordinates ***u*** = (*u*, *v*, *w*) in the field plane can be projected into the image plane using Equation 2:

(2) $$u^{\prime }=sHu$$

where *s* is a scale factor, ***u***′ are the homogeneous coordinates of the image point, ***u*** are the coordinates of that same point in the field plane and *H* is the 3 × 3 homography matrix. The determination of H allows the computation of the projections of points from one plane to the other or even the modification of the whole image for a bird’s-eye view of the scene. A common method in computer vision based on point correspondences calculates this homography matrix. We define correspondences as sets of *n* pairs of points (***u_i_***, ***u*′_*i*_**) such that a point (***u_i_***) in the field plane corresponds to ***u*′_*i*_** in the image plane. The following homogeneous system of linear equations requires a solution for H and the scale factors *s_i_* (Equation 3):

(3) $$s_{i}{u^{\prime }}_{i}=Hu_{i},(i=1,\ldots ,n)$$

The system has *n*(*d* + 1) equations and *n* + (*d* + 1)^2^ − 1 unknowns. *H* can be determined up to an overall scale factor by using *n* = *d* + 2 point correspondences as long as no more than *d* of them are collinear. For 2D planar images, this means that four point correspondences are needed, of which no more than two are collinear.

**Figure 4.**
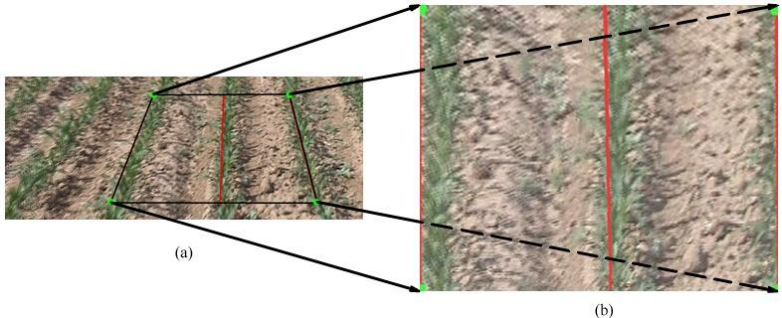
**(a)** The vertices of the trapezoid shown are used as points for the computation of the homography matrix; **(b)** They transform into a rectangle of known width (twice the crop row span) in the image plane (scaled bird’s-eye image).

These four pairs of points must be computed automatically or entered manually for the algorithm to calculate the homography matrix. In our system, points in the perspective field frames were selected among the centers of the crop rows found by the crop-row tracking algorithm (see Section 2.2). Because these sets of points cannot contain more than two collinear ones, they were chosen as the vertices of the trapezoid with the two outermost tracked crop rows as vertical sides and the two horizontal lines passing through the top and bottom centers as horizontal sides (as seen in Figure 4). The corresponding points in the transformed image were chosen to be the vertices of a rectangle of selected dimensions [Figure 4(b)]. The width of this rectangle corresponds to twice the inter-row spacing (usually approximately 0.7 m in maize crops), and the height determines the vertical scale factor of the transformation, which must be calibrated by the measurement of a known object.

After computing the homography matrix, warping the whole image by applying the inverse perspective mapping to each pixel produces a planar crop field image (or a bird’s-eye view) in which parallel rows remain parallel and, once calibrated, distances can be measured.

### 2.4. Map Generation

Because our work aims to build a map of the crop field using recorded video sequences, we need to integrate the information contained in each segmented bird’s-eye frame into a complete map of the entire field length. This map consists of a matrix of specific dimensions, with each of its elements corresponding to a cell of a given size in the real field. Moreover we must select an adequate scale factor depending on the precision and field size needed. The values of the matrix elements are determined by how many times weeds are found in the cell that the element represents. Higher values correspond to those cells where weeds were found in a larger number of frames. When a white pixel is found in the segmented frame, the matrix element corresponding to that field location (cell) is increased by one unit. Because the vehicle on top of which the camera is mounted moves forward, each new frame covers a slightly different field area, and the map’s frame of reference must be updated. The distance in pixels that the reference moves between frames depends on the speed of the vehicle, and (due to working with recorded sequences) this has been estimated using the characteristic speed of 6 km/h (1.667 m/s). Once a particular field area has been mapped, the map contains different values (ranging from zero to some certain maximum) that each refer to the number of frames containing vegetation cover (weeds and crops) in the corresponding field cell. In this manner, a higher number implies a higher level of certainty that the corresponding field cell contains vegetation.

After mapping, the matrix can be converted into a grayscale image in which higher values are lighter (white) colors and darker grey or black pixels represent lower values. Applying a threshold here retains only those cells in the map in which vegetation definitely occurs. To select an adequate value for this threshold, we analyzed the map matrix for the video sequence tested. Typical values for the matrix elements (the number of frames where weeds were present in a particular cell) ranged from 0 (no weeds in that area in any frame) to 17. To eliminate false weeds in the map due to segmentation errors in some particular frames, we selected a threshold corresponding to approximately 25% of the maximum value. Thus, any value below 4 in the map matrix was not considered in the final map. This graphical representation offers a quick view of the complete length of the field covered by the map represented in the matrix.

## 3. Results and Discussion

As previously stated, the algorithm was tested with different video sequences recorded from an autonomous tractor in an actual spraying operation (at speeds of approximately 6 km/h or 1.667 m/s). Its general performance was satisfactory, and crop rows were successfully detected and tracked for most frames in the sequences. The videos were also stabilized. Some detection errors were present due to the misidentification of weeds and crops in areas in which one of the crop rows thinned down and weeds became the major green zone, but these cases occurred in less than 7% of the frames.

As a measure of the importance of video-sequence stabilization, we measured the distance between the image horizontal center and the calculated position of the central crop row in the lower part of each frame (y = 215 pixels) for one of the sequences in which unwanted motion was more evident. In this area, the central crop row of a stabilized sequence should remain close to the horizontal center of the frame (even in the context of perspective). However, the measured deviations usually vary from 26 pixels to the left of the center to 93 pixels to the right (as shown in Table 1).

**Figure 5.**
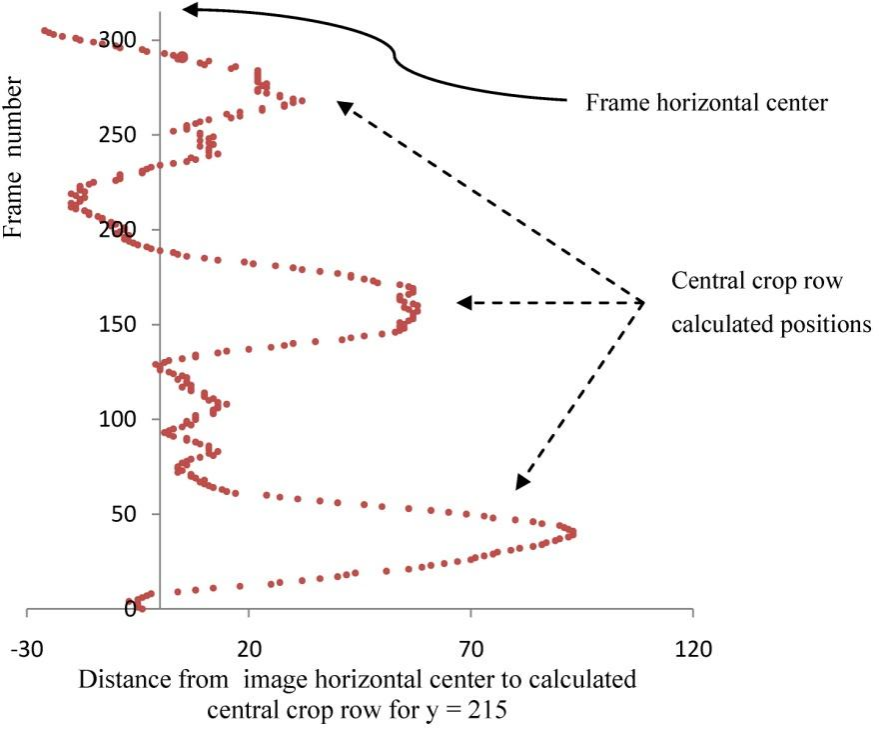
Graphical representation of the measured distance between the horizontal frame center and the calculated position of the central crop row in the lower part of the frame (y = 215) for the 306 test frames.

Figure 5 shows the measured distances for the 306 frames of the test-video sequence. The distance between the two lines is not constant but varies greatly throughout the video sequence.

The average distance of 20 pixels is significant enough to make the stabilization process meaningful, and this is even more the case when accounting for the appreciable standard deviation (28.5 pixels) and the maximum distance between lines. This maximum 93-pixel deviation accounts for 66% of the inter-row average distance (140 pixels) and places the left crop row close to the center row position. After the stabilization, the central crop row remains close to the frame’s center throughout the sequence. The stabilization of the video sequence is therefore fully justified even if it adds computational costs to the mapping process.

**Figure 6.**
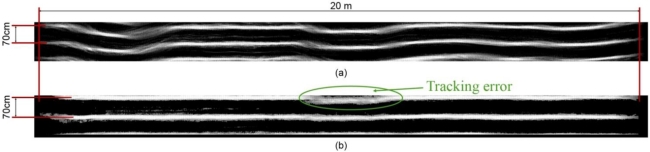
**(a)** Grayscale image of the generated field map for a video sequence without stabilization. The crop rows meander along the moving direction; **(b)** After the stabilization process, the crop rows remain straight despite the camera sway. The area where the left row twists is due to a tracking error.

**Figure 7.**
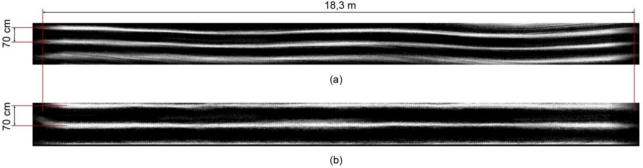
In this case, the sequence corresponds to a sunny day on a field with low weed cover. **(a)** The grayscale image of the generated map for the video sequence without stabilization depicts crop rows meandering slightly along the moving direction; **(b)** After the stabilization process, the crop rows stay completely straight despite the slight sway of the camera.

Figures 6 and 7 show the maps made before and after the stabilization process for two test video sequences that were recorded at different times of day and in different fields. Figure 6 corresponds to a crop field with low weed cover that was recorded on a partly cloudy day over rough terrain; these conditions contribute to the noticeable crop-row twisting along the moving direction. A tracking error occurred in this first sequence around the middle of the crop field. In this area, the plant density in the left crop row is reduced due to sowing errors, and some isolated weed patches were misidentified as the real crop row, causing the tracking error.

In Figure 7, the tested video sequence was taken on a smoother surface, and thus, the crop rows in the unstabilized map present less twisting [Figure 7(a)]. The rows in the stabilized map are almost completely straight [Figure 7(b)], and weed occurrences are lower than in the previous case.

**Figure 8.**
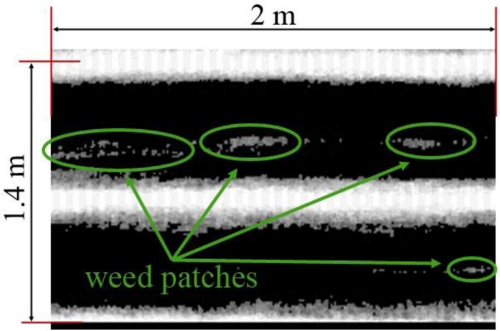
Crop area in the first video sequence in which weeds are present. Due to the large scale of the maps, a more appropriate resolution is needed in order to detect the occurrence of weeds.

Because the maps have a rather large horizontal scale factor (the maps correspond to either 18 or 20 m of terrain), the presence of weeds cannot be seen without applying the proper resolution to the obtained images. For example, Figure 8 shows a small portion of the whole field in the first test video in which weeds were detected between the crop rows.

## 4. Conclusions

Crop mapping is a crucial stage in the Precision Agriculture process. Accurate information is needed to use autonomous vehicles that apply treatments in the field or that perform other agricultural tasks. Fields must be mapped, and weeds must be precisely located. Given that the most adequate information-gathering method currently consists of cameras mounted on autonomous vehicles, some amount of instability and noise in the recorder images must be expected. To generate precise maps, these disturbances should be addressed, and the stabilization process plays an important role in this effort.

To compensate for camera motion and stabilize the sequence, many stabilization systems make use of point features that are present and that maintain a stable position in all or most of the images in the sequence. In crop fields, this problem remains important given the constant motion and slightly downward tilt of the cameras (which eliminates the horizon line in every image) and due to the absence of permanent features.

The crop-row detection and tracking algorithm presented here for video-sequence stabilization works successfully for various fields and sequences. Crop rows were detected and tracked, and the lateral camera sway and roll were removed by keeping a region of interest centered on the screen.

Trials were conducted for various sequences that were recorded in different fields at different times and under different lighting conditions, with generally promising results. The distances between some reference features, such as the central crop row and the horizontal center of the frame, remained invariant once the video sequence was stabilized. Lateral displacements of up to 66% of the inter-row spacing were suppressed.

As seen in the images, the generated maps give graphical proof of the importance of the stabilization process. Unstabilized maps present zigzagging crop rows that differ significantly from the real crop rows. Straight crops should remain straight despite the lateral sway of the camera (due to the terrain roughness), and the generated stabilized maps depict this feature for all tested video sequences.

In areas where weed infestation was high and the inter-row space was covered with green weeds, the stabilization algorithm had problems separating crops from weeds. The detection and tracking of crop rows could also be improved to deal with isolated absences of plants in the rows (sowing errors) to prevent the mischaracterization by the algorithm of the presence of weeds elsewhere in the images as crop rows. This leads to incorrect crop-row center-line calculations and to stabilization errors as well [as shown in the first map in Figure 6(b)]. However, from the test presented in this paper, we can conclude that the algorithm is robust and that to affect its performance, gaps in the crop rows (sowing errors) must be quite significant or must be coupled with appreciable camera sway.

A memory method could be developed to use the lines calculated in *good* frames as a reference or prediction in the line calculation in frames with sowing errors or with high vegetation density. This would reduce the weaknesses of the proposed approach. These modifications are high on our list of future improvements that also includes suppressing vibrations by means of a mechanical compensating device in the camera support.

## Figures and Tables

**Table 1.** Distances between the central crop row and the real center of the image on the horizontal axis for 306 frames.

**Variable**	**Value in pixels**
Distance average	20
Standard deviation	28.5
Maximum distance to the right	93
Maximum distance to the left	26
